# The Macrophage Inflammatory Proteins MIP1**α** (CCL3) and MIP2**α** (CXCL2) in Implant-Associated Osteomyelitis: Linking Inflammation to Bone Degradation

**DOI:** 10.1155/2014/728619

**Published:** 2014-03-25

**Authors:** Ulrike Dapunt, Susanne Maurer, Thomas Giese, Matthias Martin Gaida, Gertrud Maria Hänsch

**Affiliations:** ^1^Department of Orthopaedics and Trauma Surgery, University Hospital Heidelberg, Schlierbacher Landstraße 200a, 69118 Heidelberg, Germany; ^2^Department of Immunology, Heidelberg University, Im Neuenheimer Feld 305, 69120 Heidelberg, Germany; ^3^Department of Pathology, Heidelberg University, Im Neuenheimer Feld 224, 69120 Heidelberg, Germany

## Abstract

Bacterial infections of bones remain a serious complication of endoprosthetic surgery. These infections are difficult to treat, because many bacterial species form biofilms on implants, which are relatively resistant towards antibiotics. Bacterial biofilms elicit a progressive local inflammatory response, resulting in tissue damage and bone degradation. In the majority of patients, replacement of the prosthesis is required. To address the question of how the local inflammatory response is linked to bone degradation, tissue samples were taken during surgery and gene expression of the macrophage inflammatory proteins MIP1**α** (CCL3) and MIP2**α** (CXCL2) was assessed by quantitative RT-PCR. MIPs were expressed predominantly at osteolytic sites, in close correlation with CD14 which was used as marker for monocytes/macrophages. Colocalisation of MIPs with monocytic cells could be confirmed by histology. In vitro experiments revealed that, aside from monocytic cells, also osteoblasts were capable of MIP production when stimulated with bacteria; moreover, CCL3 induced the differentiation of monocytes to osteoclasts. In conclusion, the multifunctional chemokines CCL3 and CXCL2 are produced locally in response to bacterial infection of bones. In addition to their well described chemokine activity, these cytokines can induce generation of bone resorbing osteoclasts, thus providing a link between bacterial infection and osteolysis.

## 1. Introduction

Total joint replacement by endoprosthesis is a widely used procedure to restore functionality of joints in patients with osteoarthritis. Although surgery is usually safe and successful, complications may arise, particularly due to bacterial infections on and around the implant. According to the literature, the risk of infection is approximately 1 to 2% in primary arthroplasty [1–3]. Considering the ever-increasing number of prostheses which are implanted every year, infections result in high socioeconomic costs since the treatment is often prolonged and expensive [4].

The common cause of implant-associated infection is the formation of bacterial biofilms on the implant [5]. First, bacteria adhere to foreign surfaces, and then they produce and embed themselves in an extracellular substance, the name-giving “film.” Among others, bacteria in biofilms acquire a relative resistance towards many antibiotics [6, 7] (reviewed in [6, 8–12]). Therefore, extensive antibiotic treatment often fails, which makes revision surgery with tissue debridement mandatory. Because bone infections are associated with loss of bone substance; loosening of implants is a common complication and requires an exchange of the prosthesis. However, each revision surgery bears an increased risk of yet another infection.

In implant infection, staphylococci species are prevalent, but other species including enterococci or streptococci are also found and possibly also infections with multiple species [12, 13].

Bacterial biofilms elicit a profound local immune response with infiltration of neutrophils, monocytes, and T cells, associated with the local generation of proinflammatory cytokines [14, 15]. Basically, neutrophils are able to attack and destroy biofilms [16–19], but in the case of implant infections, host-defence mechanisms may be inefficient and a persistent and progressive inflammatory response might ensue, causing tissue damage and bone resorption (osteolysis) [15]. Presumably, the cytokine-rich proinflammatory environment promotes the generation of bone resorbing osteoclasts from myeloid precursor cells, but the exact mechanisms are still unclear.

In this context, the possible participation of the macrophage inflammatory proteins MIP1*α* (CCL3) and MIP2*α* (CXCL2) was assessed in this study. MIPs were initially described as chemokines, produced by monocytes or macrophages. Their participation in host defence against infection and in acute or chronic phases of inflammation is well documented. There is, however, increasing evidence for production of these cytokines by cells other than monocytes, macrophages, or neutrophils, for example, by endothelial cells, fibroblasts, neural tissue, and a variety of tumor cells (for review see [20–26]).

Both, CCL3 and CXCL2, are also described in the context of osteoclast generation and osteolysis, particularly in the mouse [27–29]. To assess their participation in implant-associated infection, we analysed gene expression and protein expression in biopsies derived from patients with implant-associated infection and for comparison in patients with aseptic loosening. The latter is an example for a sterile inflammation, which presumably is caused by the uptake of implant-derived wear particles by phagocytic cells and which also eventually leads to implant loosening [30]. Furthermore, in a series of vitro experiments, primary osteoblasts as a possible source of CCL3 and CXCL2 were assessed, as well as their role in the induction of osteoclastogenesis.

## 2. Materials and Methods

### 2.1. Patients

Patients who underwent revision surgery due to a prosthetic infection and patients suffering from aseptic loosening of a total joint replacement (articulating materials either metal-on-polyethylene or ceramic-on-polyethylene) were included in the study. Diagnosis of loosening was based on patients' complaints, clinical examination, and examination by conventional X-ray and/or CT scan.

### 2.2. Collection of Tissue and Blood Samples

From five patients with an infection and five patients with an aseptic loosening of an implant, tissue samples were taken from sites of osteolysis and for comparison from unaffected muscle. The samples were immediately placed into RNA later (Ambion Life Technologies, Darmstadt, Germany) for quantitative PCR analysis. Immediately before surgery, blood samples from patients with implant-associated osteomyelitis (*n* = 39), aseptic loosening (*n* = 22), or healthy donors (*n* = 10) were collected for gene expression analysis and ELISA (see below).

### 2.3. Histology

The tissue specimens were formalin-fixed, decalcified in ethylenediaminetetraacetic acid (EDTA), and paraffin embedded. Haematoxylin and eosin (H&E) staining was performed. The biopsies were examined, the diagnosis of an acute or chronic osteomyelitis was made, and the cellular infiltrates in particular the PMN, lymphocytes, and monocytes/macrophages were quantified. The paraffin-embedded tissue sections (3-4 *μ*m) were also used for immunohistochemical analyses. Immunostaining was performed as previously described using the avidin-biotin complex method [31]. Prior to antibody incubation, heat pretreatment in citrate buffer (pH 6.1) was performed. As primary antibody, the monoclonal mouse anti MIP-1alpha was used (R&D Systems, Darmstadt, Germany).

### 2.4. Quantitative Real-Time Polymerase Chain Reaction

From cells, mRNA was isolated with the MagnaPure-LC device using the mRNA-I standard protocol. Tissue samples were disrupted with RiboLyser devices (ThermoHYBAID, Heidelberg, Germany) containing 400 *μ*L lysis buffer from the MagnaPure mRNA Isolation kit containing 1% DTT (v/w) (ROCHE Diagnostics, Mannheim, Germany). mRNA was isolated with the MagnaPure-LC device using the mRNA-standard protocol for cells. mRNA was reversely transcribed using AMV-RT and oligo-(dT) as primer (First Strand cDNA synthesis kit, Roche, Mannheim, Germany) according to the manufactures protocol. Primer sets optimized for the LightCycler (RAS, Mannheim, Germany) were purchased from SEARCH-LC GmbH (http://www.search-lc.com/). The PCR was performed with the LightCycler FastStart DNA Sybr GreenI kit (RAS) according to the protocol provided. To control for specificity, a melting curve analysis was performed. The copy number was calculated from a standard curve, obtained by plotting known input concentrations of four different plasmids at log dilutions to the PCR-cycle number (CP) at which the detected fluorescence intensity reaches a fixed value. To correct for differences in the mRNA content, the transcript numbers were normalized according to expression of the housekeeping gene peptidylprolyl isomerase B (PPIB). Values were given as transcripts per 1000 transcripts of PPIB.

### 2.5. Bacteria

*Staphylococcus aureus* (Seattle 1945, ATCC 25923, Wesel, Germany) and* Staphylococcus epidermidis *(RP62a, ATCC 35984, Wesel, Germany) were grown overnight on a blood agar plate at 37°C (number PB5039A, Thermo Scientific, Germany, Wesel). The following day, two to three colonies were picked from the agar plate and placed in phosphate buffered saline and adjusted to 1 × 10^9^ cells/mL.

### 2.6. Isolation of Monocytes

Monocytes were isolated from the peripheral blood of healthy donors (informed consent was obtained and the institutional guidelines were observed). The blood was layered on Polymorphprep (Axis Shield, Oslo, Norway), and the monocyte fraction was recovered. Monocytes were positively selected using anti-CD14 Micro Beads (Miltenyi Biotec, Bergisch Gladbach, Germany). The procedure yielded 99.0% CD14+ cells, as assessed by cytofluorometry. The cells were seeded into 24-well dishes (NuncTM, Wiesbaden, Germany) at a concentration of 1 × 10^6^ cells/mL in RPMI containing 10% fetal calf serum, 1% glutamine, and 1% penicillin/streptomycin (medium and supplements were obtained from Gibco Life Technologies, Darmstadt, Germany).

### 2.7. Culture of Osteoblasts

Primary osteoblast cultures derived from bone marrow of patients undergoing autologous bone graft harvesting using the reamer-irrigator-aspirator (RIA) technique [32] were cultivated in osteoblast growth medium (PromoCell, Heidelberg, Germany) containing 0.1% penicillin/streptomycin (Gibco Life Technologies). Outgrowth of cells occurred usually between 4 to 8 days. Cells were subcultivated following digestion with trypsin (0.05% Trypsin-EDTA, Life Technologies, UK) for 5 minutes at 37°C and resuspended in osteoblast growth medium. After 2 days, homogenous cell layers were seen; osteoblasts were identified by expression of collagen type I and lack of markers for myeloid cells. Osteoblasts were used for a maximum of two passages. For the experiments, osteoblasts were seeded into 24-well dishes (NuncTM, Wiesbaden, Germany) at a concentration of 2 × 10^5^ cells/mL osteoblast growth medium.

### 2.8. Stimulation of Monocytes and Osteoblasts with* Staphylococcus aureus*,* Staphylococcus epidermidis*, or Lipoteichoic Acid (LTA)

For the stimulation assays, 1 × 10^6^ monocytes or 2 × 10^5^ osteoblasts were each placed in 1 mL medium (RPMI or osteoblast growth medium, resp.) into a 24-well plate. Bacteria (*S. aureus or S. epidermidis*) were added in a ratio of 20, 100, or 500 bacteria per osteoblast and incubated for 2 hours at either 4°C or 37°C. The cells were washed twice with phosphate buffered saline; then, 400 *μ*g of vancomycin was added in 1 mL culture medium for 30 minutes at 37°C. The medium was replaced by fresh medium containing 20 *μ*g vancomycin, and cells were incubated for 24 hours or 48 hours. Supernatants were collected and stored at −20°C for ELISA. The experiments with monocytes were carried out accordingly with 50 bacteria/cell. For stimulation with LTA, osteoblasts or monocytes were incubated with LTA (Sigma, Munich, Germany) for 24 to 48 h. All experiments were carried out in duplicates and repeated at least three times with cells of different patients.

### 2.9. ELISA

CCL3 and CXCL2 in cell culture supernatants and in serum samples were determined using commercially available ELISA kits according to the protocol provided by the manufacturer. The human CXCL2 Elisa kit was purchased from Hölzel Diagnostika (Cologne, Germany), the human CCL3 kit from R&D Systems (Minneapolis, USA).

### 2.10. Generation of Osteoclasts

CD14+ monocytes were seeded into 24-well dishes (NuncTM, Wiesbaden, Germany) at a concentration of 1 × 10^6^ cells per well and allowed to adhere for 24 hours in medium (RPMI, Gibco Life Technologies, Darmstadt, Germany). Then, nonadherent cells were removed by washing, and the remaining cells were incubated in RPMI supplemented with 10% FCS (PanTM Biotech, Aidenbach, Germany), 1% penicillin/streptomycin, 1% glutamine (all obtained from Gibco), and M-CSF (25 ng/mL; R&D Systems, Minneapolis, USA). For stimulation RANKL (50 ng/mL, PeproTech, Hamburg, Germany) was added or CCL3 (MIP1*α*) (R&D Systems, Minneapolis, USA) in different concentrations. Cultures were incubated at 37°C in 5% CO_2_ for up to 14 days with a change of medium and removal of nonadherent cells at day 7. For the followup differentiation, the cells were fixed with 4% PFA for 15 minutes at 37°C and identified by their morphological appearance as giant cells with multiple nuclei. Binding of FITC-labeled Phalloidin (Sigma-Aldrich; 1 : 20 dilution for 40 minutes) revealed the typical actin ring formation [33]. Nuclei were stained using DAPI (Invitrogen, Oregon, USA), (diluted 1 : 30000 for 5 minutes). Additionally, staining with an antibody against cathepsin K was performed (anti-cathepsin K (Santa Cruz Biotechnology, Heidelberg, Germany) was diluted 1 : 50), and detected with anti-mouse IgG-Cy3. Fluorescence was visualized using a Digital Fluorescence Microscope (Keyence, Neu-Isenburg, Germany). For quantification, cells were counted on images of six high-power fields and the percentage of osteoclasts in relation to the total cell number was calculated.

### 2.11. Western Blot of Whole Cell Lysates

Following stimulation with CCL3 or RANKL (Santa Cruz Biotechnology, Heidelberg, Germany), cells (3 × 10^6^) were lysed with RIPA buffer (Santa Cruz Biotechnology, Santa Cruz, USA) and mixed with SDS-Gel sample buffer and separated by SDS-polyacrylamide gel (12%) electrophoresis, followed by blotting to a PVDF membrane (Millipore, Eschborn, Germany). The antibody to NFATc1 was purchased from Santa Cruz Biotechnology (Heidelberg, Germany), and as secondary antibody peroxidase-labelled anti-mouse IgG (Jackson Immuno Research, Pennsylvania, USA) was used. For detection, Amersham ECL plus Western Blotting Detection System (GE Healthcare Limited, Munich, Germany) was used.

### 2.12. Statistical Analysis

Differences between groups were calculated using Mann-Whitney test using Origin 9.0 software. Significance level was determined as *P* < 0.05.

## 3. Results

### 3.1. Expression of the Macrophage Inflammatory Proteins CCL3 and CXCL2 in Tissue of Patients with Infection and with Aseptic Loosening

**Figure 1:**
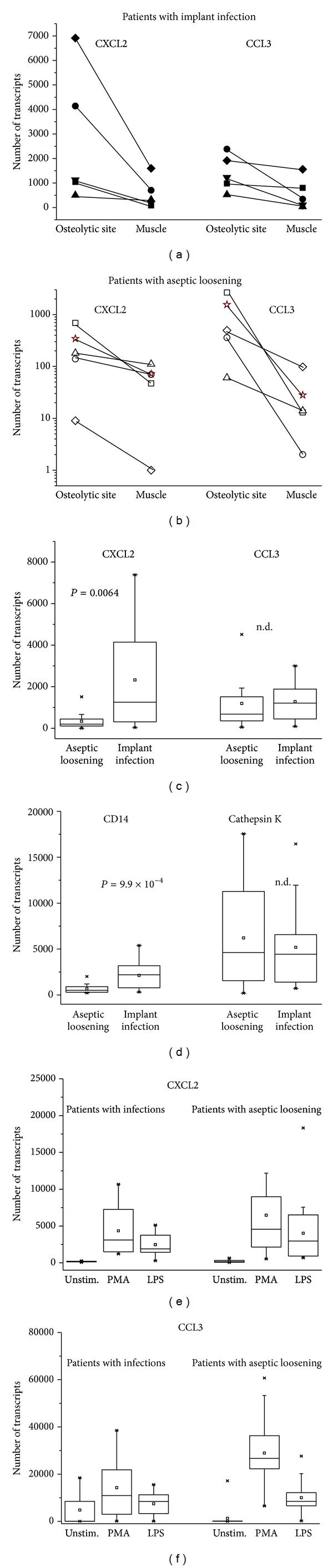
Gene expression of CXCL2 and CCL3 in tissue and blood of patients with implant-associated infection or aseptic loosening. Gene expression of CXCL2 and CCL3 was determined by qRT-PCR in tissue of patients with infection (a) or with aseptic loosening (b). Samples were collected from osteolytic sites and for comparison from muscle. Each symbol represents one patient. (c) Gene expression at osteolytic sites was compared between patients with implant infection (*n* = 6) and patients with aseptic loosening (*n* = 14), as was expression of CD14 and cathepsin K (d). The groups were analysed by Mann-Whitney test, and the respective *P* values are given. (e, f) Gene expression was analysed in blood samples obtained from patients with infection and those with aseptic loosening. Basic expression of CXCL2 and CCL3 did not differ between the groups, and cells could be stimulated to a similar extent.

Gene expression of CCL3 and CXCL2 was determined in tissues collected during surgery. Samples from osteolytic sites were taken and for comparison from unaffected muscle. Gene expression varied among the patients, but, in all, expression was higher in tissue from osteolytic sites compared to muscle. CXCL2 expression was also higher in patients with infection compared to patients with aseptic loosening, whereas expression of CCL3 did not differ between patients with infection and those with aseptic loosening (data summarised in Figure 1).

**Figure 2:**
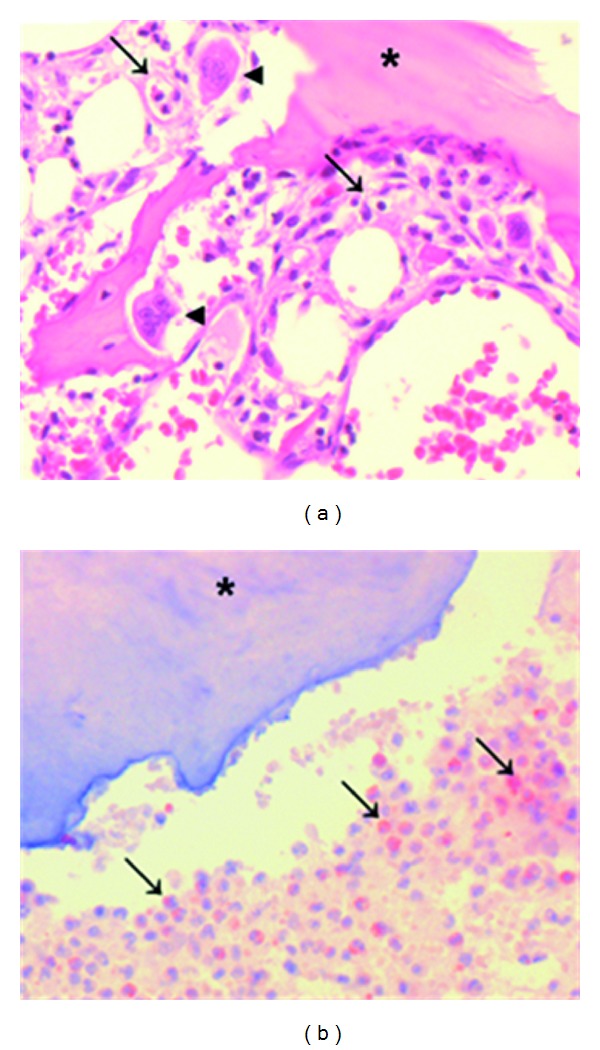
Biopsy of a patient with infection and massive osteolysis: (a) HE staining shows degraded bone (star) and osteoclasts (arrow head) in the immediate vicinity. Moreover, neutrophils and mononuclear cells are seen (arrows). (b) Specific staining for CCL3 indicated by brownish coloring is mostly associated with mononuclear cells (arrows).

Immunohistological examination confirmed the presence of CCL3 positive inflammatory cells particularly neutrophils, lymphocytes, and monocyte/macrophages. Osteoclasts were seen in the proximity of CCL3 positive inflammatory cells in the eroded bone (an example for a patient with infection and osteolysis is shown in Figure 2). By analysing gene expression of CD14, infiltration of mononuclear cells into affected sites could be confirmed, with higher expression of CD14 at osteolytic sites compared to muscle (2132.9 ± 1487.9 CD14 gene transcripts versus 864.6 ± 512.5 in muscle in patients with infection; 671.7 ± 479.2 versus 316.6 ± 189.9 in patients with aseptic loosening) and higher expression in patients with infection compared to tissue from aseptic loosening (Figure 1). Expression of cathepsin K, a marker for osteoclasts and hence of osteolysis, did not differ between the two patient groups (Figure 1).

In the peripheral blood, CCL3 was found in 8 of 14 patients with aseptic loosening (51.5 to 92.8 pg/mL) and in 5 of 34 patients with an infection (47.5 to 118.8 pg/mL); CXCL2 was not detected in sera of patients with aseptic loosening and in 5 of 34 patients with an infection (ranging from 9.4 to 514.5 pg/mL). Of note, these were not the same patients as those with measurable CCL3 serum concentrations. In serum of healthy donors neither CCL3 nor CXCL2 were found. Gene expression of CXCL2 and CCL3 in peripheral blood cells did not differ between the two patient groups, and peripheral blood cells responded essentially similar to stimulation with either PMA or LPS (Figures 1(e), and 1(f)).

### 3.2. Synthesis of CCL3 and CXCL2 by Osteoblasts

**Figure 3:**
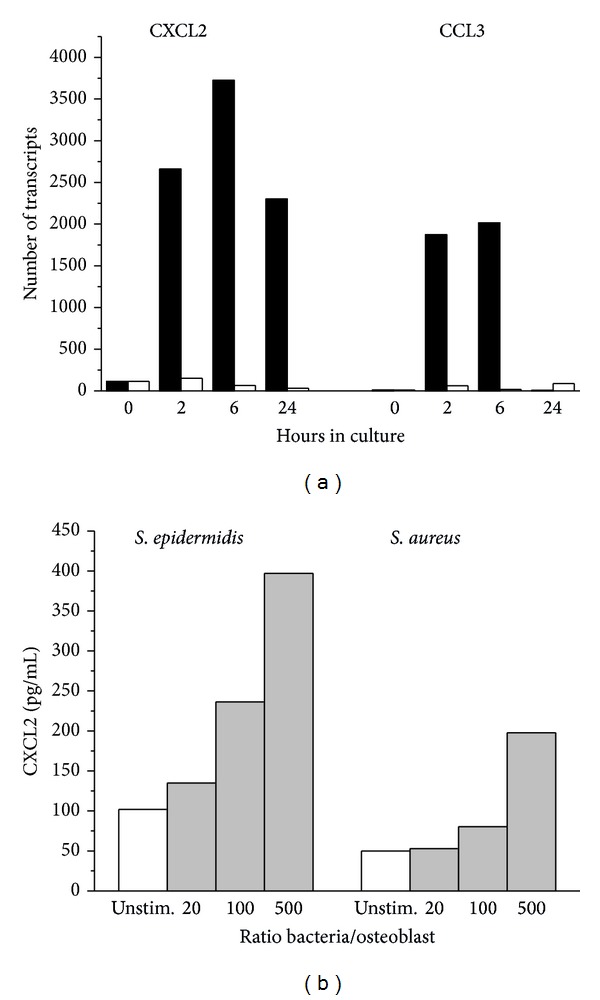
CXCL2 and CCL3 synthesis by cultivated osteoblast. (a) Gene expression of CXCL2 and CCL3 following stimulation of osteoblasts with LTA (1 *μ*g/mL) for the time indicated. (b) Release of CXCL2 into the supernatant determined following stimulation of osteoblasts with either* S. epidermidis *or* S. aureus* in three doses (given as ratio of bacteria per osteoblasts) (one of three independent experiments giving essentially similar results is shown).

Infiltrating monocytes are a likely source of cytokines, but also osteoblasts are known to produce and release cytokines in response to infectious agents ([34, 35]). To follow up on that issue, primary osteoblasts were cocultivated with LTA as an example for a bacteria-derived entity. Within 2 to 6 hours, an increase in gene expression of CCL3 and CXCL2 was seen (Figure 3(a)). In another set of experiments, osteoblasts were cultivated with either* S*.* epidermidis *or* S. aureus* for 2 hours. Bacteria were then killed and cell culture was continued for 24 hours or 48 hours. MIPs were released into the supernatant depending on the number of bacteria present (representative experiment in Figure 3(b)). On average,* S. epidermidis* when added in a relation of 500 bacteria/cell induced CXCL-2 production of 22014.75 ± 12815.2 pg/mL,* S.aureus *13966 ± 6128.36 pg/mL. For unstimulated cells, 6608.2 ± 3412.2 pg/mL was determined (values are the mean ± SD of three experiments with cells derived from different patients).

CCL3 release was only seen with 500* S. epidermidis*/cell and amounted to 71.44 ± 27.3 pg/mL versus 0 in unstimulated cells.* S. aureus*—in similar bacteria to cell ratio—did not elicit a CCL3 response.

Of note, when osteoblasts were exposed to the bacteria at 4°C as opposed to 37°C, CCL3 and CXCL2 release was within the same range (data not shown). Also, there was no significant difference between cultivation for 24 h and 48 h.

As expected, monocytes released both, CCL3 and CXCL2, into the supernatant. Both bacterial strains were equally efficient (on average 317385 pg/mL of CCL3 and 31890 pg/mL of CXCL2 were released) and again there was no major difference between 37° and 4°C (data not shown).

### 3.3. Induction of Osteoclast Formation by CCL3

**Figure 4:**
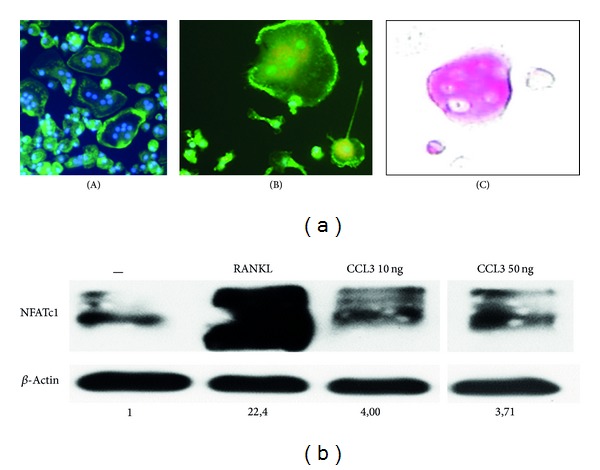
Generation of osteoclasts by CCL3. (a) Monocytes were cultivated with CCL3 (10 ng/mL); at day 18, giant cells with multiple nuclei (blue) and *β* actin in a ring-like structure were seen (A). These cells also expressed cathepsin K (B red) and TRAP (C). (b) Stimulation with CCL3 induced the induction of NFATc1. A Western blot of cell lysates derived from untreated cells (-) or from cells incubated with RANKL or CCL3 is shown. The loading of the gel was controlled and normalised using *β* actin; the numbers indicate the increase over untreated cells (for technical reasons, sample CCL3 50 ng had run in a distant lane and was cut and added).

To link generation of MIPs to osteolysis, the effect of CCL3 on the differentiation of monocytes towards osteoclasts was assessed. Generation of osteoclasts over time was monitored microscopically. After 14 to 18 days in culture, giant cells with multiple nuclei appeared, expressing cathepsin K and TRAP, enzymes characteristic of osteoclasts (example in Figure 4(a)). Because osteoclast generation depends on an extended activation and* de novo* synthesis of NFATc1 [36, 37], lysates of CCL3 treated monocytes were analysed by Western blotting. Enhancement of NFATc1 was seen, though to a lesser extent when compared to RANKL-treated cells (Figure 4(b)).

## 4. Discussion

Tissue samples of patients with implant-associated infections revealed gene expression of CCL3 and CXCL2 at sites of osteolysis, exceeding the amount detected in unaffected muscle tissue. Expression of CD14, a marker of infiltrating mononuclear cells, followed essentially the same pattern, suggesting that in accordance with data in the literature CCL3 and CXCL2 are predominately produced by macrophages (reviewed in [23]). In patients with aseptic loosening basically a similar expression pattern of CXCL2, CCL3, and CD14 was seen, though transcript numbers for CXCL2 and CD14 were lower compared to those seen in infected tissue. Aseptic loosening is a sterile inflammation presumably caused by prosthesis-derived wear particles which activate primarily myeloid cells [30, 38, 39]. This possibly accounts for the similar cytokine expression pattern. The presence of very potent stimulators of myeloid cells in infectious cases (particularly bacteria-derived lipopolysaccharides or lipoteichoic acid) could explain the more prominent reaction seen in bacterial infection. Moreover, neutrophils, which are found primarily in infected but rarely in aseptic tissue, could contribute to CCL3 and CXCL2 production and cause a more pronounced inflammatory response. Differences in the extent of the inflammatory response could explain why aseptic loosening usually progresses slower in some patients, 10 to 20 years after primary arthroplasty [39]. Eventually, however, the extent of bone degradation is rather similar, compatible with our data, showing similar transcripts for cathepsin K, a parameter for osteoclast generation.

The fact that CXCL2 gene expression in infected tissue was enhanced compared to tissue of patients with aseptic loosening, but that of CCL3 was not, suggests that its production is differently regulated or involves cells others than macrophages. Indeed, CCL3, for example, is also produced by a subset of CD8 + T cells [40, 41], or endothelial cells [42]. Fibroblasts produce both, CCL3 and CXCL2, [43], as do neutrophils [44] after stimulation.

Important for our studies was the possible production of these cytokines by osteoblasts [28, 45], a fact we were able to confirm by exposing cultivated osteoblasts to staphylococci or lipoteichoic acid (LTA), a major component of the bacterial cell wall. LTA stimulated both, CCL3 and CXCL2 expression, whereas the bacteria induced predominantly CXCL2, suggesting different signalling pathways. LTA most likely activates the osteoblasts via toll-like receptor 2, whereas staphylococci express in addition to LTA also other surface molecules which may mediate binding and activation of target cells. Among those is protein A on* S. aureus* which binds to the tumor necrosis factor *α* receptor on osteoblasts [46, 47]. Others include fibronectin-binding proteins which are expressed by bacteria and mediate binding to surface-associated fibronectin on osteoblasts [48–50]. Because* S. aureus* uses a different binding site on fibronectin as* S. epidermidis*, different signalling pathways are triggered, which could explain that synthesis of CXCL2 is stimulated by* S. aureus*, but that of CCL3 is not. Activation of CXCL2 synthesis occurred even when the osteoblasts were exposed to bacteria at 4°C, indicating that the mere contact of bacteria to cell surface receptors is sufficient to trigger the cells and ruling out phagocytosis as a crucial signal.

Enhanced local cytokine production can result in enhanced serum concentrations, and serum cytokines can serve as biomarkers to monitor disease activity. Elevated serum concentrations of MIPs were found in various chronic inflammatory diseases, including rheumatoid arthritis or systemic lupus erythematosus [51–55], as well as in experimentally induced infection in mice [56, 57]. Differentiating between aseptic implant loosening and implant failure due to an infection can be difficult, because the standard laboratory parameters for infection (serum CRP concentration or leukocyte count) can be negative in infectious cases and thus misleading. Taking the different therapeutic approaches in aseptic and infectious cases into consideration, the search for a reliable preoperative marker has become crucial in orthopaedic surgery. We therefore assessed whether CCL3 and CXCL2 in serum samples could be useful. However, neither the CCL3 and CXCL2 protein concentrations in serum, nor the gene expression by peripheral blood cells differed between aseptic and infectious cases. Most likely, in contrast to the above mentioned systemic diseases, the expression and effects of CCL3 and CXCL2 are exhibited exclusively locally, with no “spill-over” into the peripheral blood.

Locally generated CCL3 and CXCL2 could contribute as chemoattractants to the infiltration of cells; CXCL2 is a major chemokine for neutrophils, which could account for the neutrophil infiltration into infected tissues since expression of CXCL2 is enhanced in these patients as opposed to aseptic cases; CCL3 attracts predominantly CD8 + T cells, in line with the shift of the CD4/CD8 ratio towards CD8+ in the cellular infiltrate in implant-associated infection [58].

Aside from attracting immune cells, CXCL2 and CCL3 could have an additional function in implant loosening. MIPs have been shown to participate in osteoclast generation in patients with multiple myeloma and the expression correlated with bone degradation [59–61]. Moreover, in vitro studies and various rodent models provided proof of a connection between MIP-production and bone degradation [62–65].

In a series of in vitro studies, we were now able to show that CCL3 induced the differentiation of CD14+ monocytes to osteoclasts, which might be relevant for bone degradation in osteomyelitis, because peripheral blood monocytes are known to be osteoclast precursors [66].

Osteoclast differentiation induced by CCL3 was associated with NFATc1 synthesis, in line with the effect of RANKL, a well-known and major inducer of osteoclastogenesis [67, 68].

Under our experimental conditions, CCL3 was less effective when compared to RANKL, which, however, not necessarily diminishes their role in osteoclast generation in vivo. The relevance of CCL3 and CXCL2 in the process of bone resorption was shown in mouse experimental models [27].

## 5. Conclusion

In conclusion, our study demonstrates that, under infectious conditions, but also in sterile inflammation, CXCL2 and CCL3 are produced locally. Because MIPs may contribute to osteoclast generation, these findings provide a link between local inflammation and bone resorption which further leads to implant loosening. Colocalisation with CD14 suggests synthesis by cells of the monocyte/macrophage lineage, but, because osteoblasts can also produce MIPs when appropriately stimulated, it is feasible that they initiate the inflammatory response by recruiting leukocytes. This in turn creates a proinflammatory environment which results in a progressive course of the disease and leads to extensive tissue damage, as well as loss of bone.
